# Single-cell analysis of COPD: Microenvironmental interactions driven by immune-fibrotic imbalance

**DOI:** 10.1371/journal.pone.0361580

**Published:** 2026-10-26

**Authors:** Hong Jiang, Wenxing Wang, Lu Wang, Jiaojian Wu, Jianjiang Luo, Qi Yan, Xueli Gong, Jing Liang

**Affiliations:** 1 School of Nursing, Xinjiang Medical University, Urumqi, Xinjiang, China; 2 Department of Neurology, The Second Affiliated Hospital, Xinjiang Medical University, Urumqi, Xinjiang, China; 3 School of Basic Medical Science, Xinjiang Medical University, Urumqi, Xinjiang, China; 4 Institute of Medical Sciences of Xinjiang Medical University, Urumqi, Xinjiang, China; 5 School of Public Health, Xinjiang Medical University, Urumqi, Xinjiang, China; 6 Medical Records Management Department, The Affiliated Taian City Central Hospital of Qingdao University, Tai’an, China; 7 Department of Infectious Diseases, Hospital of Traditional Chinese Medicine, Xinjiang Medical University, Urumqi, Xinjiang, China; 8 Department of Pathophysiology, School of Basic Medical Science, Xinjiang Medical University, Urumqi, Xinjiang, China; 9 Chief Technologist, /Master’s Supervisor, The Sixth Affiliated Hospital of Xinjiang Medical University, Urumqi, Xinjiang, China; UT Health San Antonio: The University of Texas Health Science Center at San Antonio, UNITED STATES OF AMERICA

## Abstract

Chronic obstructive pulmonary disease (COPD) is a respiratory disease with a high degree of cellular heterogeneity and molecular complexity, characterized pathologically by persistent airflow limitation and an inflammatory-tissue remodeling cascade involving multiple cell types. Traditional transcriptomics lack the resolution to precisely define cell subpopulation roles in COPD. Single-cell RNA sequencing (scRNA-seq) overcomes this limitation by enabling high-resolution analysis of cell-type-specific mechanisms in COPD. A total of 101,009 cells from three scRNA-seq datasets were integrated to profile COPD-related molecular features and cell interactions at single-cell resolution. Analysis identified four major cell types and 17 distinct cell subtypes. Cell–cell communication analysis revealed a significant reduction in signaling activity of macrophages and fibroblasts, whereas certain immune cells exhibited enhanced signaling, suggesting that immune dysregulation plays a pivotal role in COPD pathogenesis. Further analysis focused on macrophage-dominant myeloid cells and fibroblasts, uncovering their critical roles in inflammation modulation and fibrosis progression. Meanwhile, this study also identified 19 chemicals that may influence COPD progression by modulating cellular gene expression. This study provides an important molecular basis for unraveling the cell-cell interaction network of COPD and elucidating the mechanism of each cell subpopulation in the disease process.

## 1. Introduction

Chronic obstructive pulmonary disease (COPD) is a chronic respiratory condition characterized by persistent airflow limitation. The pathology of COPD includes airway inflammation, destruction of alveolar structure (emphysema), and pulmonary fibrosis, all of which contribute to the continual deterioration of lung function [1, 2]. Although COPD has complex and multifactorial etiologies, long-term cigarette smoking and environmental exposures are considered the major risk factors [3–5]. However, the precise molecular mechanisms and the interactions among different cell types contributing to COPD pathogenesis are still not fully elucidated.

In recent years, genetic studies of COPD have made significant breakthroughs through genome-wide association studies (GWAS), identifying several key loci such as HHIP, FAM13A, DSP, AGER, CHRNA3/5, and TGFB2 [6–13]. These findings reveal complex regulatory mechanisms beyond α1-antitrypsin deficiency. For instance, FAM13A and DSP have been shown to directly influence the Wnt/β-catenin signaling pathway [14], which facilitates lung injury repair by promoting cell proliferation, differentiation, and stem cell renewal [15]. Additionally, both FAM13A and TGF-β2 are involved in regulating epithelial–mesenchymal transition (EMT) [16, 17]. Despite the unprecedented insights provided by GWAS into the genetic basis of COPD, several limitations remain. First, GWAS mainly identifies disease-associated genetic loci, but these loci are mostly located in non-coding or regulatory regions, making their biological functions difficult to define directly. Most importantly, although GWAS can be used to uncover associations between genetic variants and diseases, its results often face the dilemma of “missing cell-type localization,” meaning it is a challenge to determine the functional role of a specific genetic variant within a particular lung cell type.

The development of Single-cell RNA sequencing (scRNA-seq) technology has provided a methodological breakthrough for COPD research. With its high-resolution capabilities, scRNA-seq effectively addresses the limitations of GWAS by enabling the characterization of cellular heterogeneity and the discovery of underlying molecular mechanisms in COPD [18, 19]. This technique not only enables researchers to accurately characterize the gene expression profiles of different cell types in the lungs [20], but also provides new perspectives for exploring the dynamics of specific cells in disease states. By applying scRNA-seq technology, researchers are able to more deeply analyze the complex regulatory network of COPD and advance the functional analysis of genetic variation to the level of specific cell types and the disease microenvironment in which they reside. Recent studies utilizing scRNA-seq have uncovered the involvement of various cell populations in COPD. For example, ciliated cells and CD8 + T cells are prominent cell types associated with emphysema. The former plays a key role in maintaining mucociliary clearance, while the latter is closely linked to airway inflammation and alveolar destruction [21, 22]. Dysfunctional alveolar epithelial cells contribute to both structural damage and pulmonary fibrosis through impaired regeneration and pro-fibrotic phenotype switching [23, 24]. Myeloid cells, such as macrophages, participate in disease progression by modulating inflammatory responses and tissue repair mechanisms [25], whereas fibroblasts contribute to fibrosis through remodeling of the extracellular matrix (ECM) [26, 27].

Although advances in scRNA-seq have significantly deepened our understanding of COPD, current research remains heavily focused on epithelial cells—particularly in relation to epithelial barrier dysfunction, ciliary impairment, and abnormal expansion of secretory cells [18, 23, 26, 28–30]. However, key questions regarding the interplay between myeloid cells and fibroblasts remain in the early stages of investigation. Notably, systematic comparisons between COPD patients and healthy individuals at single-cell resolution are still lacking. The functional characteristics of relevant cell subpopulations and their dynamic remodeling under pathological conditions also require further exploration. These gaps in knowledge impede a thorough grasp of COPD pathogenesis and obstruct the advancement of cell subtype–specific therapeutic strategies. Moreover, the impact of environmental exposures and chemical compounds on the expression of COPD-associated genes, and their potential roles in disease progression, remain important yet underexplored areas of research.

Given the unique advantages of scRNA-seq in resolving cellular heterogeneity, this study integrates multiple publicly available scRNA-seq datasets and employs clustering analysis, functional annotation, and pseudotime trajectory analysis to systematically explore the molecular characteristics and intercellular interactions in COPD and healthy controls at single-cell resolution. Furthermore, the functional changes and interaction mechanisms of key myeloid cell populations—especially macrophages—and fibroblasts were further analyzed. By combining our findings with chemical–gene interaction analyses, we identify candidate compounds that may modulate COPD-related gene expression. Ultimately, by characterizing the heterogeneity of cellular subpopulations in COPD patients and their subpopulation-specific transcriptional profiles, this study highlights COPD-associated cellular and molecular features that may inform future investigations of cell-state heterogeneity, environmental exposure-related mechanisms, and chemical-gene associations in COPD.

## 2. Methods

### 2.1. scRNA-seq dataset information

In this study, publicly available scRNA-seq datasets, including lung tissue samples from both COPD patients and healthy subjects, were collected from the Gene Expression Omnibus (GEO, https://www.ncbi.nlm.nih.gov/geo/). The datasets selected were GSE173896 (5 COPD and 5 healthy), GSE279570 (11 COPD and 7 healthy) and GSE167295 (3 COPD and 0 healthy) (Table 1).

**Table 1:** Data set information.

Data Type	GEO ID	Source	Platform	COPD patients	Healthy subjects	Cell number
scRNA-seq	GSE173896	Lung	Illumina HiSeq X	5	5	41,053
	GSE279570		Illumina NovaSeq 6000	11	7	64,764
	GSE167295		Illumina NextSeq 500	3	0	11,465
Total				19	12	117,282

### 2.2. Data preprocessing

In this study, the Seurat R package (v4.3.3) [31] was used for the preprocessing of the three scRNA-seq datasets. Dataset-specific filtering criteria were applied to ensure the accuracy of downstream analyses. During the experiment, cells with more than 300 expressed genes and a mitochondrial gene proportion less than 10% were retained. Each sample was classified based on clinical status (COPD or healthy control), and corresponding annotation information was incorporated.

To support quality control and doublet screening, a preliminary log-normalization was applied, with gene expression levels standardized via ScaleData solely for these pre-processing objectives. Potential doublets were identified and eliminated using DoubletFinder (v2.0.3) [32], with detailed detection parameters provided in Supplementary Methods 4. A total of 45,721 COPD cells and 55,288 healthy cells were retained for analysis.

During data integration, the raw UMI counts of each dataset—following doublet removal-were individually normalized using the SCTransform method [33]. To improve computational efficiency, the glmGamPoi algorithm [34] was employed to accelerate the transformation process. The PrepSCTIntegration function was then used for secondary preprocessing of the SCTransform-normalized gene expression matrices. Next, the FindIntegrationAnchors function was applied to detect anchor gene pairs across datasets within a low-dimensional space. Finally, the IntegrateData function was applied to integrate the datasets, ensuring compatibility and consistency across different data sources. Additionally, to further minimize the influence of experimental variation, the Harmony algorithm was applied to correct for batch effects and optimize the quality of integration.

### 2.3. Cell clustering

To perform clustering analysis on the integrated scRNA-seq data, this study first performed principal component analysis (PCA) to extract key features. We used the ElbowPlot function from the Seurat R package to visualize the standard deviations of the principal components (PCs). The number of PCs selected for subsequent clustering analysis was determined based on the inflection point of the plot, along with the criterion that the cumulative explained variance exceeds 90%. After determining the principal components, the clustering results were further evaluated using the clustree package (v0.5.1) [35] to select the optimal resolution parameter. Specifically, clustree constructs a hierarchical clustering tree and evaluates the stability of clustering at different resolutions, thereby identifying the clustering resolution that best reflects the true structure of cell types. Based on the evaluation results from clustree, the optimal resolution parameter was chosen to balance clustering resolution and biological significance, which was then used for subsequent cell type annotation and differential expression analysis (DEA). Next, we used the FindNeighbors function from Seurat to construct a k-nearest neighbors (KNN) graph based on the low-dimensional PCA space, followed by clustering of cells using the Louvain algorithm [36] via the FindClusters function, to identify cell groups with similar gene expression profiles. Detailed parameter settings for these steps are provided in Supplementary Methods 1.

### 2.4. Cell annotation

To accurately annotate the cell types of each cell cluster, this study first identified marker genes specific to each cell cluster. Differential expression analysis (DEA) was performed using the FindAllMarkers function in the Seurat package. During the analysis, only up-regulated genes were considered (only.pos = TRUE), with a minimum fraction of cells expressing the gene set to 25% (min.pct = 0.25). Additionally, a log fold-change threshold of 0.5 (logfc.threshold = 0.5) was applied. P-values were adjusted using the Benjamini-Hochberg (BH) method, and only genes with an adjusted p-value < 0.05 were considered statistically significant. The selected markers were then used to annotate cell types, using the top 10 markers ranked by log2FC as the basis for annotation. Manual annotation of each cell cluster was performed by combining the classical cell type marker genes from the CellMarker database [37], and corresponding cell type labels were assigned.

### 2.5. Differential expression and functional enrichment analysis

To identify differentially expressed genes (DEGs) between the COPD groups and healthy control (CTL) groups, the FindMarkers function from the Seurat package was applied to the normalized raw expression matrix, Statistical analysis based on Wilcoxon rank sum test. As an initial filtering step, only genes present in at least 25% of the cells (min.pct = 0.25) were retained, and a logFC threshold of > 0.5 (logfc.threshold = 0.5) was applied to ensure that the analyzed genes exhibited significant changes in expression. Significance was determined using the BH procedure, with an adjusted p-value cutoff of < 0.05. Subsequently, the clusterProfiler package (v4.6.2) [38] was employed to perform Gene Ontology (GO) enrichment analysis, aiming to uncover the possible biological roles of these differentially expressed genes. The enrichGO() function was applied to evaluate the involvement of these genes in biological processes (BP), molecular functions (MF), and cellular components (CC). The enrichment outcomes were subsequently visualized to reveal the functional significance of these genes across different biological categories.

### 2.6. Cell communication analysis

To examine how various cell types interact, CellChat (v2.1.2) [39] was used to conduct cell communication analysis independently for COPD patients and healthy controls. First, the identifyOverExpressedGenes and identifyOverExpressedInteractions functions were used to identify significantly expressed ligand-receptor pairs, and the computeCommunProb function was applied to calculate the communication probability between cells, thereby constructing a cell communication network. Next, the computeCommunProbPathway function was used to calculate the overall transmission probability of signaling pathways, and the aggregateNet function was employed to calculate the communication intensity between cell types. Finally, various visualization methods were utilized to intuitively display the major communication patterns and key signaling pathways between cells, revealing the cell communication characteristics associated with COPD (see Supplementary Methods 2 for detailed analytical procedures).

### 2.7. Pseudotime analysis

To explore the developmental pathways and differentiation potentials of cells, pseudotime analysis was conducted using Monocle3 (v1.3.7) [40–43]. Initially, the preprocess_cds function was employed to normalize the data and perform preliminary dimension reduction. Subsequently, dimensionality-reduced data were visualized using Uniform Manifold Approximation and Projection (UMAP) through the reduce_dimension function. Following this, cell clustering was carried out using the Leiden algorithm with the cluster_cells function, and the cellular trajectory was constructed using the learn_graph function. Lastly, the order_cells function was applied to determine the trajectory’s root state, enabling the reconstruction of pseudotime progression throughout cellular development.

### 2.8. Chemical-gene interaction analysis

To identify possible upstream regulators of crucial genes, interactions between chemicals and genes were analyzed using data from the Comparative Toxicogenomics Database (CTD) [44]. First, DEGs were extracted for each cell type and screened for the top 10 genes that were up-regulated and down-regulated in each cell, and then the CTD database API was used to search for chemicals associated with these genes, focusing on compounds that either up-regulated the expression of these up-regulated genes or down-regulated the expression of these down-regulated genes. Subsequently, chemical-gene associations were classified based on the type of interaction (activation or inhibition), and the potential roles of these chemicals in COPD were assessed. Furthermore, we integrated disease association information provided by the CTD to filter chemicals that are highly relevant to the study subjects, with the aim of identifying potential environmental or drug intervention targets. Detailed parameter settings for these steps are provided in Supplementary Methods 5.

## 3. Results

### 3.1. scRNA-seq dataset analysis

To resolve the molecular signature of COPD at single-cell resolution, this study selected three publicly available lung scRNA-seq datasets. After quality control, 45,721 cells from 19 COPD patients and 55,288 cells from 12 healthy controls were included. Initial UMAP visualization of the datasets revealed significant batch effects (Supplementary Figures 2 and 3). To address this, the Harmony algorithm was applied to the Principal Component Analysis (PCA) embeddings of the integrated data to correct for technical variations. Subsequent visualization confirmed the effective removal of batch effects (Supplementary Figures 4 and 5), followed by cell clustering and differential expression analysis. Finally, cell type annotation was performed using the CellMarker [37] databas. In total, four major cell types (Fig 1B) and 17 subtypes (Fig 1D) were identified from 101,009 cells. Specifically, the identified cell types included: Immune cells (B cell, CD4 + T cell, CD8 + T cell, Dendritic cell, Macrophages, Mast cell, Monocytes, NK cell, Plasma cell, Stem cell); Epithelial cells (ATO, AT1, AT2, Ciliated cell); Endothelial cells; and Stromal cells (Fibroblasts, Smooth muscle cell). The dot plot of marker genes for these cell types is shown in Supplementary Figure 6. Visualization of the top 10 highly variable genes (Fig 1A) showed that these genes are mainly involved in key changes related to immunoglobulin synthesis, airway barrier function and immune cell migration. In addition, we examined the distribution ratios of the four major cell types in COPD patients and healthy controls. In the COPD group, immune cells constituted the predominant proportion of the total cell population (Fig 1C). This observation highlights the fundamental abundance of immune cells within the lung tissue. Specifically, in the context of COPD, this persistent immune dominance aligns with the disease’s characteristic chronic inflammatory status, providing potential clues to its pathogenesis.

**Fig 1:**
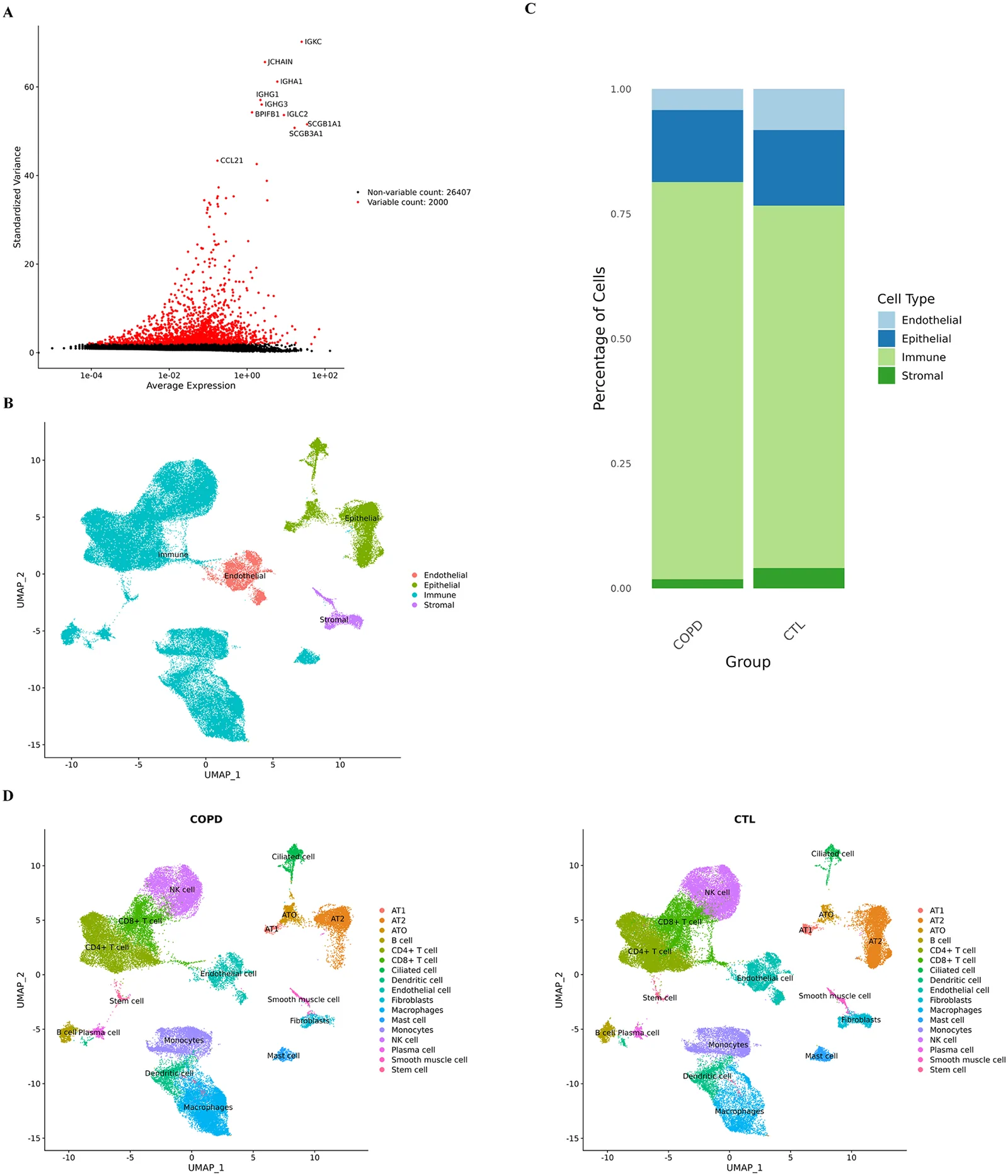
scRNA-seq Analysis Reveals Differences in Cell Types and Subpopulations Between COPD and CTL Groups. A. Scatter plot of highly variable genes. This plot shows the top 10 genes with the highest standard deviation. B. UMAP dimensionality reduction plot of cell type annotation results. This plot displays the overall distribution of first-level cell annotations across all samples. C. Stacked bar plot of cell type proportions. This graph shows the relative percentages of the different cell types between the two groups of cells. D. UMAP dimensionality reduction plot of cell subtype annotation results. This plot presents the UMAP distribution of second-level cell annotations in both the COPD and CTL groups.

### 3.2. Cell communication analysis

To gain deeper insights into the intercellular communication patterns in COPD patients versus healthy controls, a comprehensive analysis of cell-cell interactions was performed based on the scRNA-seq data. By analyzing scatter plots of signal emission and reception strengths across different cell types, it was observed that macrophages, fibroblasts, AT1, and smooth muscle cells in the COPD group exhibited lower inferred communication activity than those in the CTL group (Fig 2A). This indicates that the functions of these cell types may be suppressed under the pathological conditions of COPD. Further detailed investigation of their signaling networks suggested changes in inferred signaling activities, particularly COLLAGEN and FN1 signaling, in interactions involving macrophages and fibroblasts (Fig 3A–B). COLLAGEN and FN1 signaling are closely associated with extracellular matrix organization, cell–matrix interactions, and tissue remodeling. Notably, both COLLAGEN and FN1 signaling showed stable patterns across individual datasets, supporting the reproducibility of these ECM-associated communication features. These differences suggest that signaling interactions involving macrophages and fibroblasts may be associated with coordinated extracellular matrix remodeling and tissue-repair transcriptional features in the COPD microenvironment. Additionally, the cell-cell communication network visualized by chord diagrams revealed that interactions in the COPD group were predominantly attenuated, particularly those involving macrophages and fibroblasts (Fig 2B). This reflects the possible imbalance in the COPD condition. Finally, the overall cell communication quantity and signal strength were quantitatively compared through bar graphs (Fig 2C). The results indicated that the overall communication network in the COPD group exhibited a redistribution pattern.

**Fig 2:**
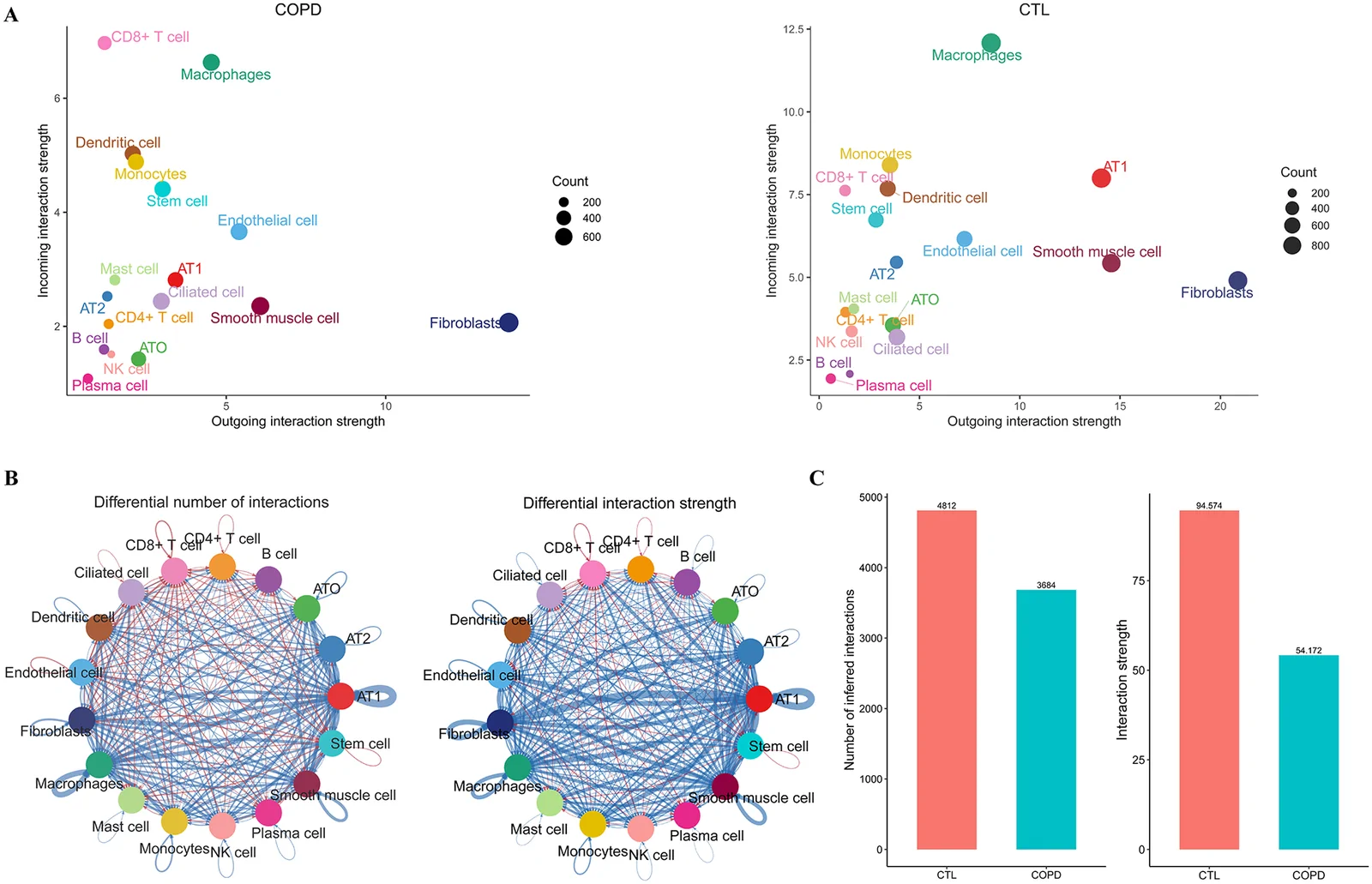
Cell Communication Analysis between COPD Patients and Healthy Controls. **A. Overall distribution of cell communication strength**. The x-axis shows the signal emission strength of cells, while the y-axis indicates the strength of signal reception. **B.** Vi**sualization of cell communication network quantity and strength**. The left panel illustrates the variation in cell communication counts between the COPD and CTL groups. Red represents elevated cell communication frequency or intensity in the COPD group compared to the CTL group, while blue signifies a reduction. **C. Bar plot of overall communication quantity and strength**. The left bar graph represents the number of total cells communicating between the two groups, and the right bar graph represents the signal strength between the two groups.

**Fig 3:**
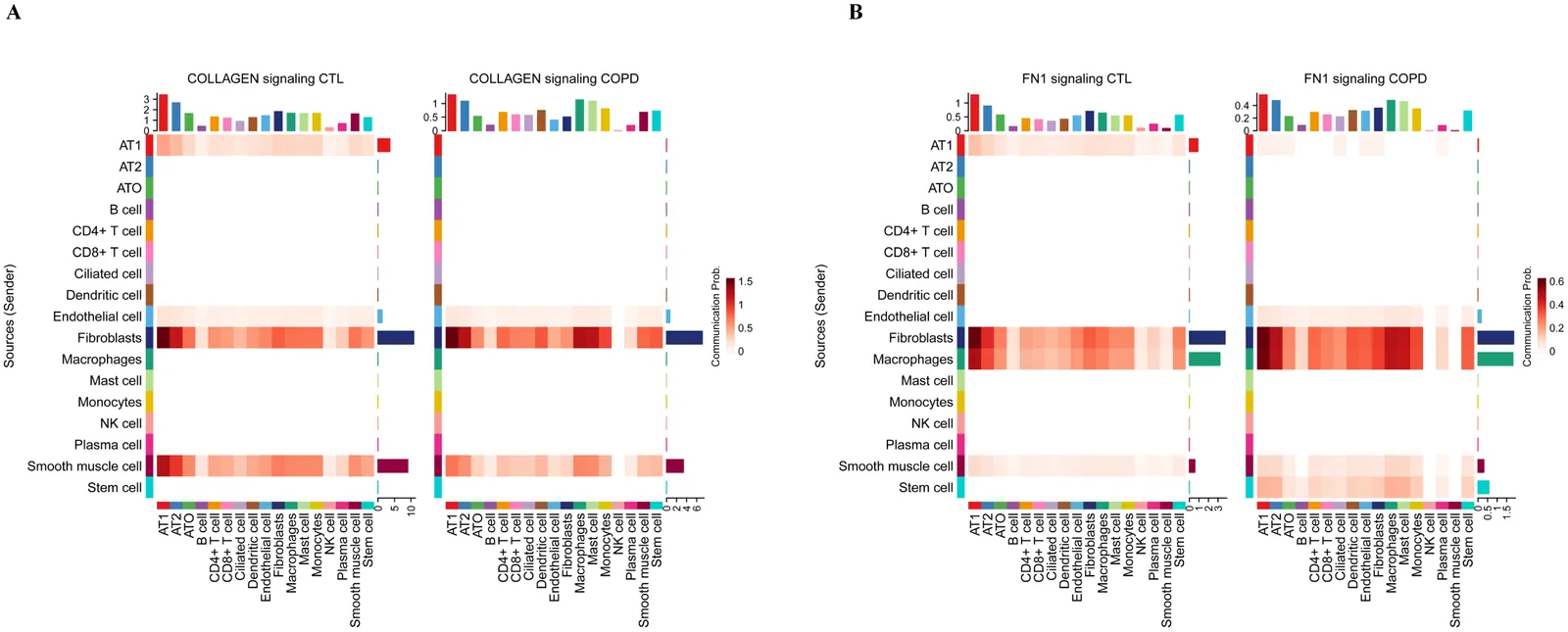
Comparative Analysis of Cellular Sources of Signaling Pathways between COPD Patients and Healthy Controls. The map shows the ligand-receptor interaction patterns of signaling pathways such as COLLAGEN and FN1 across different cell types. The intensity of the color represents signal strength.

In summary, these findings suggest that the cell communication network in COPD patients undergoes complex remodeling, and the changes in the signaling functions of macrophages and fibroblasts may play a critical role in the processes of inflammation and tissue remodeling.

### 3.3. Myeloid cell analysis

To identify the transcriptomic changes of macrophages and other myeloid cells associated with COPD, this study reconstructed the subpopulations of monocytes, dendritic cells, and macrophages from the raw data, ultimately identifying 10 functionally specialized subpopulations (Fig 4A). Specifically, two monocyte subpopulations were identified: classical monocytes and non-classical monocytes; four dendritic cell subpopulations were identified: cDC1, cDC2, migratory DCs, and plasmacytoid DCs; and four macrophage subpopulations were identified: alveolar macrophages, alveolar macrophages CCL3 + , alveolar macrophages MT-positive, and interstitial macrophages perivascular. The dot plot of marker genes for these cell types is shown in Supplementary Figure 7. The pseudotime trajectory, reflecting transcriptional dynamics, is shown in Fig 4B. Notably, alveolar macrophages and non-classical monocytes exhibited relatively high proportions within the myeloid landscape (Fig 4C). Integrating this observation with pseudotime analysis, we found that these two populations possessed the highest transcriptomic diversity, suggesting a broad spectrum of functional states.

**Fig 4:**
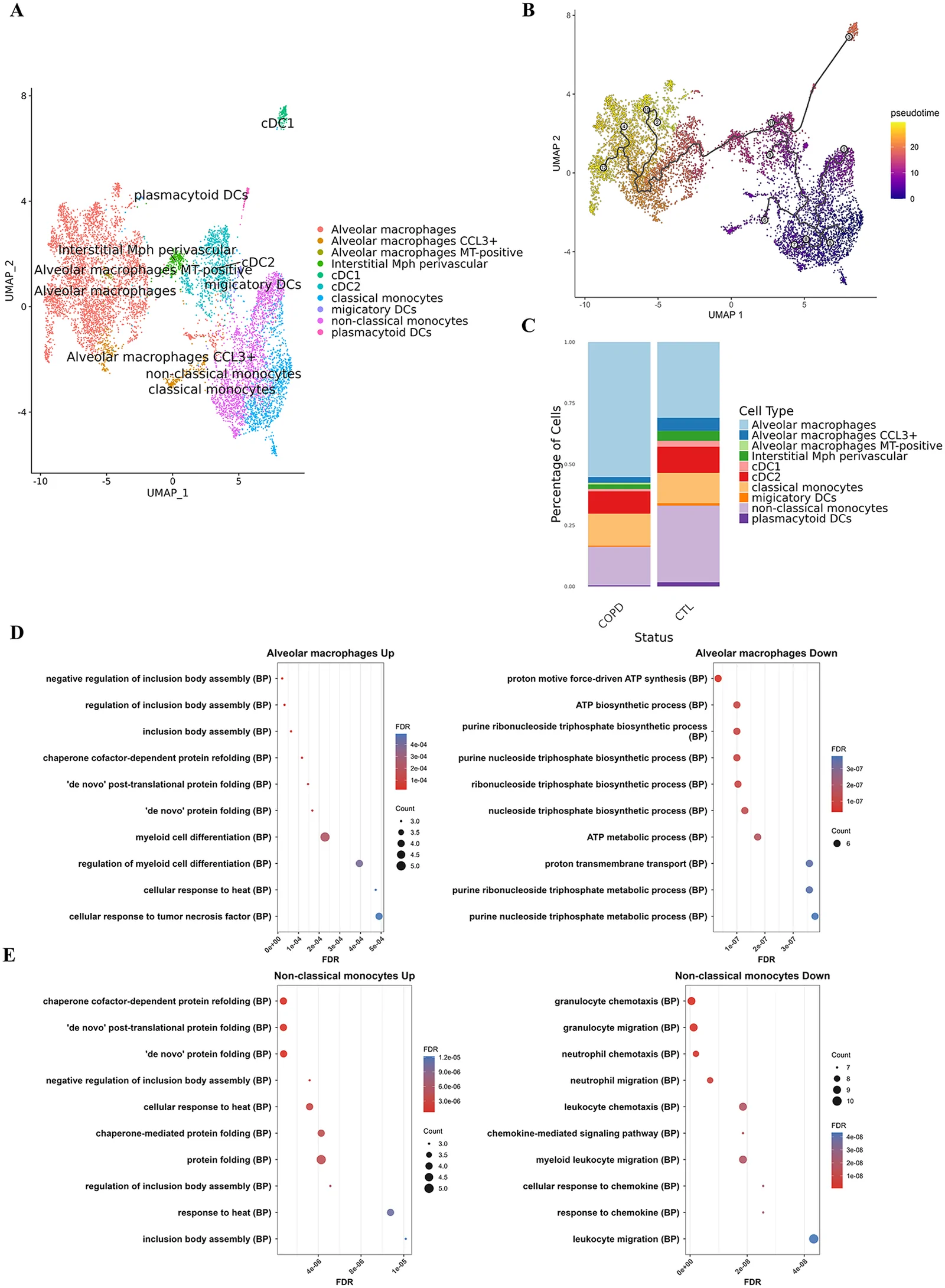
Analysis Results of Myeloid Cells. **A. Annotation of Cell Subtypes**. UMAP downscaling map showing the distribution and clustering of all cells in low-dimensional space. **B**. **Cell Differentiation Trajectory Analysis**. The pseudotime analysis constructs a trajectory of cell state transitions. The UMAP plot shows the pseudotime progression with a color gradient, and gray circles mark key nodes with corresponding labels. **C**. **Stacked Bar Plot of Cell Type Composition by Group**. The x-axis shows the groups (e.g., COPD and CTL), while the y-axis shows the proportion of each type of cell. Different colors indicate different cell subpopulations, allowing for the evaluation of compositional variations in major cell types between groups. **D**. **Functional Enrichment Bubble Plot**. This plot shows the significantly enriched Gene Ontology (GO) pathways for upregulated and downregulated DEGs in alveolar macrophages from the COPD group compared to the CTL group. **E**. **Similar to D**, this plot shows the significantly enriched GO pathways for upregulated and downregulated DEGs in non-classical monocytes from the COPD group compared to the CTL group.

To deeply elucidate the molecular characterization of the key subpopulations, differential expression gene analyses were systematically carried out between the COPD and CTL groups, targeting two important cell populations: alveolar macrophages (COPD:2807, CTL:1070) and non-classical monocytes (COPD:800, CTL:1085). The results showed 43 DEGs in COPD alveolar macrophages (17 upregulated/26 downregulated) (S1 Data) and 46 DEGs in non-classical monocytes (15 upregulated/31 downregulated) (S2 Data).

GO functional enrichment analysis revealed that the 17 upregulated genes in COPD alveolar macrophages were mainly enriched in biological processes related to altered myeloid differentiation, protein folding, and inflammatory or heat stress responses. Specifically, enriched terms included myeloid cell differentiation, regulation of myeloid cell differentiation, chaperone cofactor-dependent protein refolding, “de novo” post-translational protein folding, “de novo” protein folding, cellular response to heat, cellular response to tumor necrosis factor, and regulation of inclusion body assembly (Fig 4D; S3 Data). These results suggest that COPD alveolar macrophages exhibit transcriptional features associated with altered differentiation-related programs and activation of protein-folding and stress-response pathways. The enrichment of protein-folding and inclusion-body-related processes may reflect changes in protein homeostasis, whereas the enrichment of cellular response to tumor necrosis factor is consistent with an inflammation-associated macrophage transcriptional state in COPD. In contrast, the 26 downregulated genes in COPD alveolar macrophages were primarily enriched in energy metabolism-related biological processes, including proton motive force-driven ATP synthesis, ATP biosynthetic process, nucleoside triphosphate biosynthetic process, ATP metabolic process, purine nucleoside triphosphate metabolic process, and proton transmembrane transport (Fig 4D; S4 Data). These findings indicate that downregulated genes in COPD alveolar macrophages are associated with ATP synthesis, proton transmembrane transport, and nucleotide energy metabolism. The reduced enrichment of these processes may reflect altered bioenergetic activity in COPD alveolar macrophages.

For COPD non-classical monocytes, the 15 upregulated genes were mainly enriched in biological processes associated with protein homeostasis, protein folding, and heat stress responses, including chaperone cofactor-dependent protein refolding, “de novo” post-translational protein folding, “de novo” protein folding, chaperone-mediated protein folding, protein folding, response to heat, cellular response to heat, and regulation of inclusion body assembly (Fig 4E; S5 Data). These results suggest that COPD non-classical monocytes display enhanced transcriptional features related to protein folding and heat stress responses. In contrast, the 31 downregulated genes in COPD non-classical monocytes were mainly enriched in immune cell migration and chemokine-response-related biological processes, including granulocyte chemotaxis, granulocyte migration, neutrophil chemotaxis, neutrophil migration, leukocyte chemotaxis, leukocyte migration, myeloid leukocyte migration, chemokine-mediated signaling pathway, cellular response to chemokine, and response to chemokine (Fig 4E; S6 Data). These findings suggest that COPD non-classical monocytes may exhibit reduced transcriptional programs related to chemokine responsiveness, leukocyte migration, and neutrophil recruitment.

Finally, based on the Hallmark gene sets from the MSigDB database, GSVA scores were calculated for each cell subpopulation (Fig 5). In alveolar macrophages, the GSVA scores were significantly enriched in pathways related to ADIPOGENESIS, Fatty_acid_metabolism, and Protein_secretion, suggesting that alveolar macrophages may undergo metabolic reprogramming and enhance immune factor secretion, further promoting intercellular interactions and local immune modulation. In contrast, non-classical monocytes were significantly enriched in inflammatory pathways such as: IL6_JAK_STAT3_SIGNALING, INFLAMMATORY_RESPONSE, suggesting that non-classical monocytes may be involved in the regulation of immuno-inflammation, which further supports the role of this cell in the persistent inflammatory environment. The present analysis suggests coordinated COPD-associated transcriptional alterations in alveolar macrophages and non-classical monocytes, highlighting protein homeostasis and chemokine signaling as candidate pathways for future mechanistic investigation.

**Fig 5:**
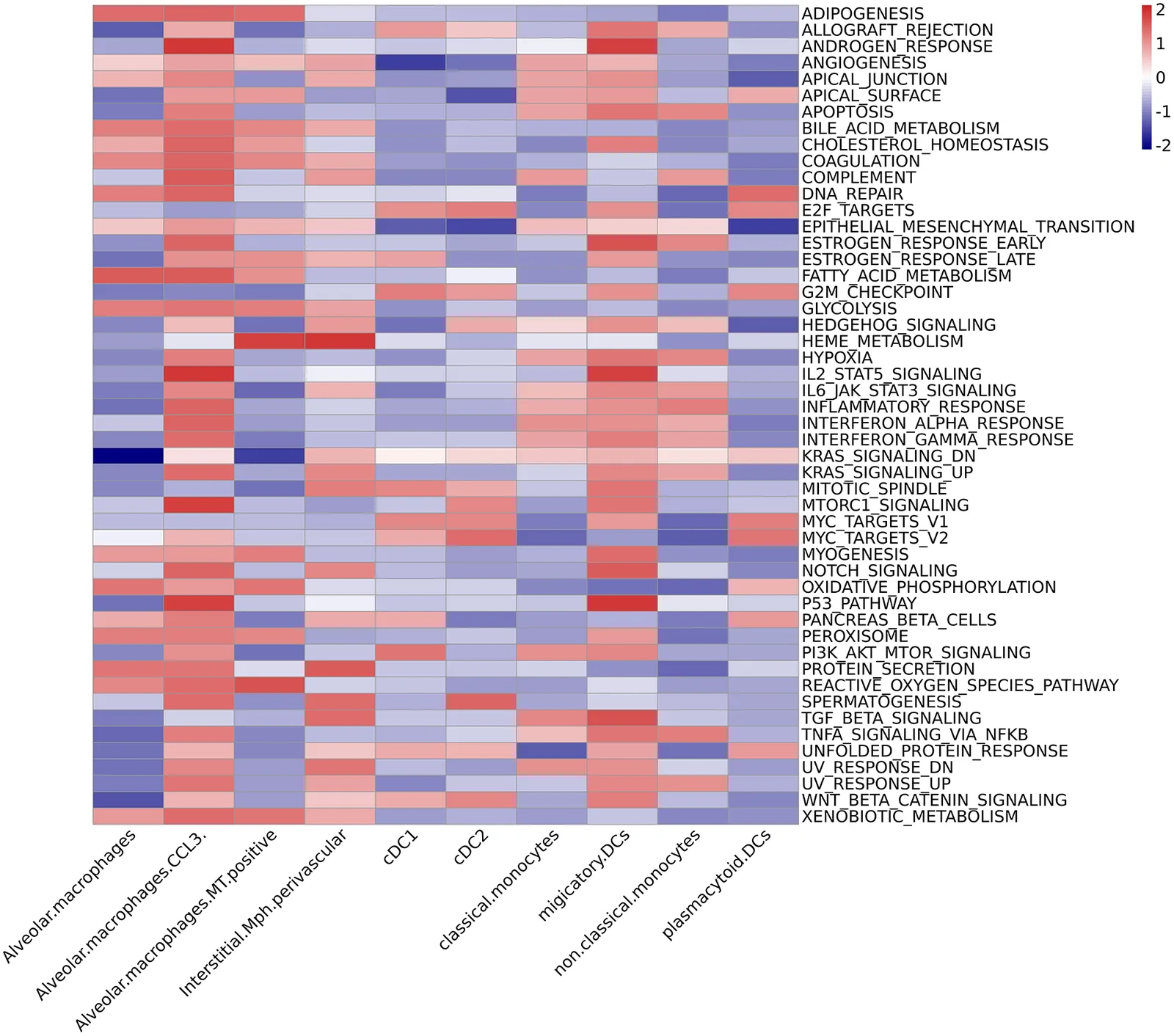
Signaling pathway activity analysis across cell subpopulations. The heatmap displays the gene set variation analysis (GSVA) scores of 50 classical signaling pathways across different cell subpopulations, with the color gradient representing the activity levels of the pathways.

### 3.4. Fibroblast analysis

To identify transcriptomic alterations in fibroblasts associated with COPD, this study performed subclustering of fibroblasts from the original dataset and ultimately identified four functionally specialized subpopulations (Fig 6A), namely: ACTA2 + , PLA2G2A + , TCF21 + , and others. The dot plot of marker genes for these cell types is shown in Supplementary Figure 8. The pseudotime trajectory, reflecting transcriptional dynamics, is shown in Fig 6B. Meanwhile, trajectory analysis revealed that both TCF21+ and ACTA2 + subpopulations exhibited the most extensive transcriptomic diversity. As illustrated in the subpopulation composition profile (Fig 6C), the presence of these distinct cell states indicates that these lineages possess a broad spectrum of functional states.

**Fig 6:**
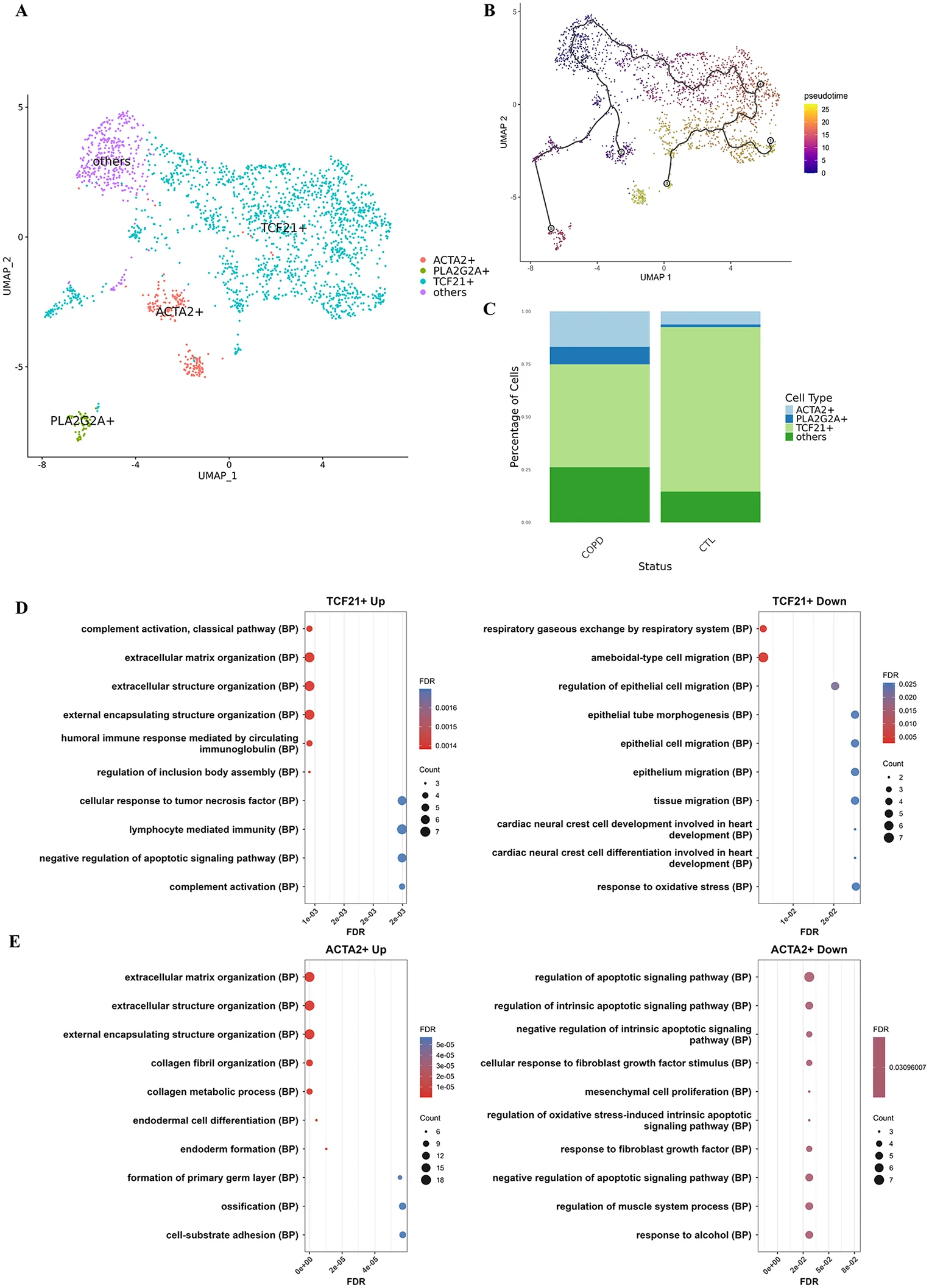
Analysis results of fibroblasts. **A. Annotation of Cell Subtypes**. The UMAP dimensionality reduction plot shows the distribution and clustering of all cells in a low-dimensional space, with each dot representing a single cell. Different colors indicate different fibroblast subpopulations. **B**. **Cell Differentiation Trajectory Analysis**. Pseudotime analysis was used to construct the trajectory of fibroblast state transitions. The color gradient in the UMAP plot represents the progression of pseudotime, while gray circles mark key nodes, each labeled with a corresponding identifier. **C**. **Stacked Bar Plot of Cell Type Composition by Group**. The x-axis represents the comparison groups (e.g., COPD vs. CTL), and the y-axis indicates the proportion of each fibroblast subpopulation. Different colors represent different cell types, which allows for evaluating differences in fibroblast composition between groups. **D**. **Functional Enrichment Bubble Plot**. This plot displays significantly enriched GO terms linked to upregulated and downregulated DEGs in TCF21 + cells from the COPD group compared to the CTL group. **E. Similar to D**, this plot shows significantly enriched GO terms for upregulated and downregulated DEGs in ACTA2 + cells from the COPD group compared to the CTL group.

To further elucidate the molecular characteristics of key subgroups, differential gene expression analysis was conducted between COPD and CTL for two critical cell populations: TCF21+ (COPD: 265, CTL: 1296) and ACTA2+ (COPD: 91, CTL: 103) fibroblasts. The results showed that 70 DEGs were identified in TCF21 + cells from COPD patients (41 upregulated/29 downregulated) (S7 Data), and 110 DEGs were identified in ACTA2 + cells (66 upregulated/44 downregulated) (S8 Data).

GO enrichment analysis indicated that the 41 upregulated genes in TCF21 + cells from COPD patients were mainly enriched in biological processes related to extracellular matrix organization, immune activation, apoptosis-related regulation, and cellular stress responses. Specifically, enriched terms included extracellular matrix organization, extracellular structure organization, external encapsulating structure organization, complement activation, complement activation classical pathway, humoral immune response mediated by circulating immunoglobulin, lymphocyte-mediated immunity, negative regulation of apoptotic signaling pathway, cellular response to tumor necrosis factor, and regulation of inclusion body assembly (Fig 6D; S9 Data). These findings suggest that COPD TCF21 + cells exhibit transcriptional features associated with ECM organization, immune-response-related transcriptional features, TNF-related inflammatory responses, and altered apoptosis-related regulation. In contrast, the 29 downregulated genes in TCF21 + cells were primarily enriched in biological processes related to epithelial migration, tissue migration, morphogenesis, respiratory gas exchange, and oxidative stress response. Representative terms included respiratory gaseous exchange by respiratory system, ameboidal-type cell migration, regulation of epithelial cell migration, epithelial cell migration, epithelium migration, tissue migration, epithelial tube morphogenesis, cardiac neural crest cell development involved in heart development, cardiac neural crest cell differentiation involved in heart development, and response to oxidative stress (Fig 6D; S10 Data). These results suggest that COPD TCF21 + cells may show reduced transcriptional programs related to epithelial/tissue migration, morphogenesis-associated processes, and oxidative-stress responses. The enrichment of epithelial migration-related terms among downregulated genes may be associated with altered epithelial repair-related transcriptional features in the COPD microenvironment.

For ACTA2 + cells, the 66 upregulated genes in COPD patients were predominantly enriched in ECM organization, collagen-related remodeling, developmental differentiation, and cell-substrate adhesion. Enriched terms included extracellular matrix organization, extracellular structure organization, external encapsulating structure organization, collagen fibril organization, collagen metabolic process, endodermal cell differentiation, endoderm formation, formation of primary germ layer, ossification, and cell-substrate adhesion (Fig 6E; S11 Data). These findings suggest that COPD ACTA2 + cells exhibit enhanced transcriptional features related to ECM organization and collagen remodeling, potentially suggesting a matrix-producing fibroblast-like transcriptional phenotype in COPD. Consistently, the gene expression heatmap showed increased expression of collagen-related genes, including COL1A1 and COL3A1, in COPD patients compared with healthy controls (Fig 7A; Supplementary Figure 1). The 44 downregulated genes in ACTA2 + cells were mainly enriched in biological processes related to apoptosis regulation, fibroblast growth factor response, mesenchymal cell proliferation, oxidative stress-induced apoptotic signaling, and muscle system regulation. Representative terms included regulation of apoptotic signaling pathway, regulation of intrinsic apoptotic signaling pathway, negative regulation of intrinsic apoptotic signaling pathway, regulation of oxidative stress-induced intrinsic apoptotic signaling pathway, cellular response to fibroblast growth factor stimulus, response to fibroblast growth factor, mesenchymal cell proliferation, regulation of muscle system process, negative regulation of apoptotic signaling pathway, and response to alcohol (Fig 6E; S12 Data). These results suggest that COPD ACTA2 + cells may exhibit altered transcriptional programs related to intrinsic apoptotic signaling, FGF-associated responses, mesenchymal proliferation-related transcriptional programs, and muscle-related regulatory processes.

**Fig 7:**
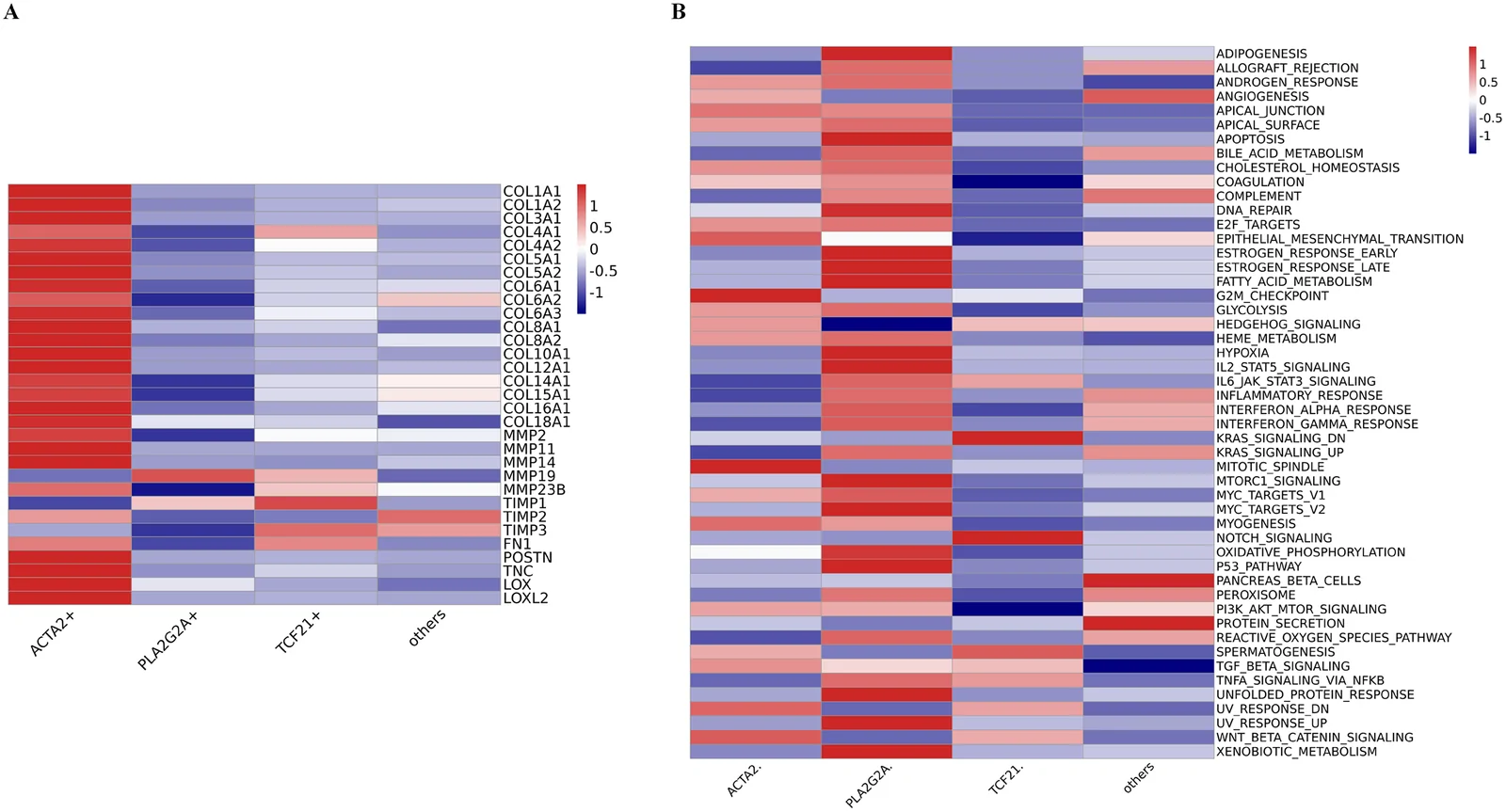
Heatmap Analysis of Fibroblast Subpopulations in COPD. A. **Expression Profiles of ECM-Related Genes**. The heatmap displays the expression patterns of ECM-related genes across fibroblast subpopulations in COPD patients. The color gradient reflects gene expression levels, revealing potential functional differences among subpopulations in the context of inflammation. **B. Pathway Activity Analysis**. The heatmap shows GSVA scores for 50 hallmark signaling pathways across fibroblast subpopulations, with the color gradient indicating the relative activity level of each pathway.

Finally, based on the Hallmark gene sets from the MSigDB database, this study performed GSVA for each cell subpopulation (Fig 7B). In TCF21 + cells, GSVA was significantly enriched in pathways such as KRAS_SIGNALING_DN and NOTCH_SIGNALING, suggesting that these cells may play important roles in regulating cell proliferation, differentiation, and tissue remodeling. In ACTA2 + cells, significant enrichment was observed in EPITHELIAL_MESENCHYMAL_TRANSITION (EMT), G2M_CHECKPOINT, MITOTIC_SPINDLE, and MYOGENESIS pathways, indicating that these cells may be involved in tissue remodeling, cell cycle regulation, and differentiation. Notably, the enrichment of EMT suggests that ACTA2 + cells may contribute to aberrant tissue remodeling and pathological fibrosis. This analysis characterizes the heterogeneity of fibroblast subpopulations in COPD and suggests potential coordinated transcriptional patterns that may inform future studies of fibroblast subgroup-specific functions.

### 3.5. Chemical–gene interaction analysis

To identify key chemical factors that may regulate cellular functions in the context of COPD, this study constructed chemical–gene interaction networks based on the CTD. Specifically, the analysis focused on disease-associated DEGs identified by comparing COPD patients versus healthy controls within each cell subtype (alveolar macrophages, non-classical monocytes, ACTA2 + cells, and TCF21 + cells). By systematically screening the top 10 upregulated and downregulated DEGs with the most significant expression changes in each cell subset, we identified potential regulatory chemical compounds associated with these genes (S13–S14 Data).

UpSet analysis revealed partial overlap among chemical compounds that promote the expression of upregulated DEGs across the four cell subsets (Fig 8A), and similarly, compounds that suppress downregulated DEGs exhibited shared features (Fig 8B). The horizontal bar plots at the top quantify the degree of overlap in chemical–gene interactions across cell subsets, with red bars indicating the number of chemical compounds shared among multiple subsets. Quantitative analysis identified 62 chemical compounds that significantly promoted the expression of upregulated DEGs in the four key subsets—alveolar macrophages, non-classical monocytes, TCF21 + cells, and ACTA2 + cells—while 37 compounds were found to specifically suppress the expression of downregulated DEGs within these subsets. Notably, we identified 19 chemical compounds with dual regulatory effects (Fig 8C), including: Acetaminophen, Benzo(a)pyrene, Bisphenol A, Cadmium Chloride, Estradiol, Methylmercuric Chloride, Silicon Dioxide, Sodium Arsenite, Trichostatin A, Valproic Acid, Rotenone, Tetrachlorodibenzodioxin, Tobacco Smoke Pollution, 7,8-Dihydro-7,8-dihydroxybenzo(a)pyrene 9,10-oxide, Hydrogen Peroxide, Particulate Matter, Doxorubicin, Formaldehyde, and Vorinostat. These dual-function compounds were found to both significantly upregulate the expression of upregulated DEGs and effectively suppress the expression of downregulated DEGs.

**Fig 8:**
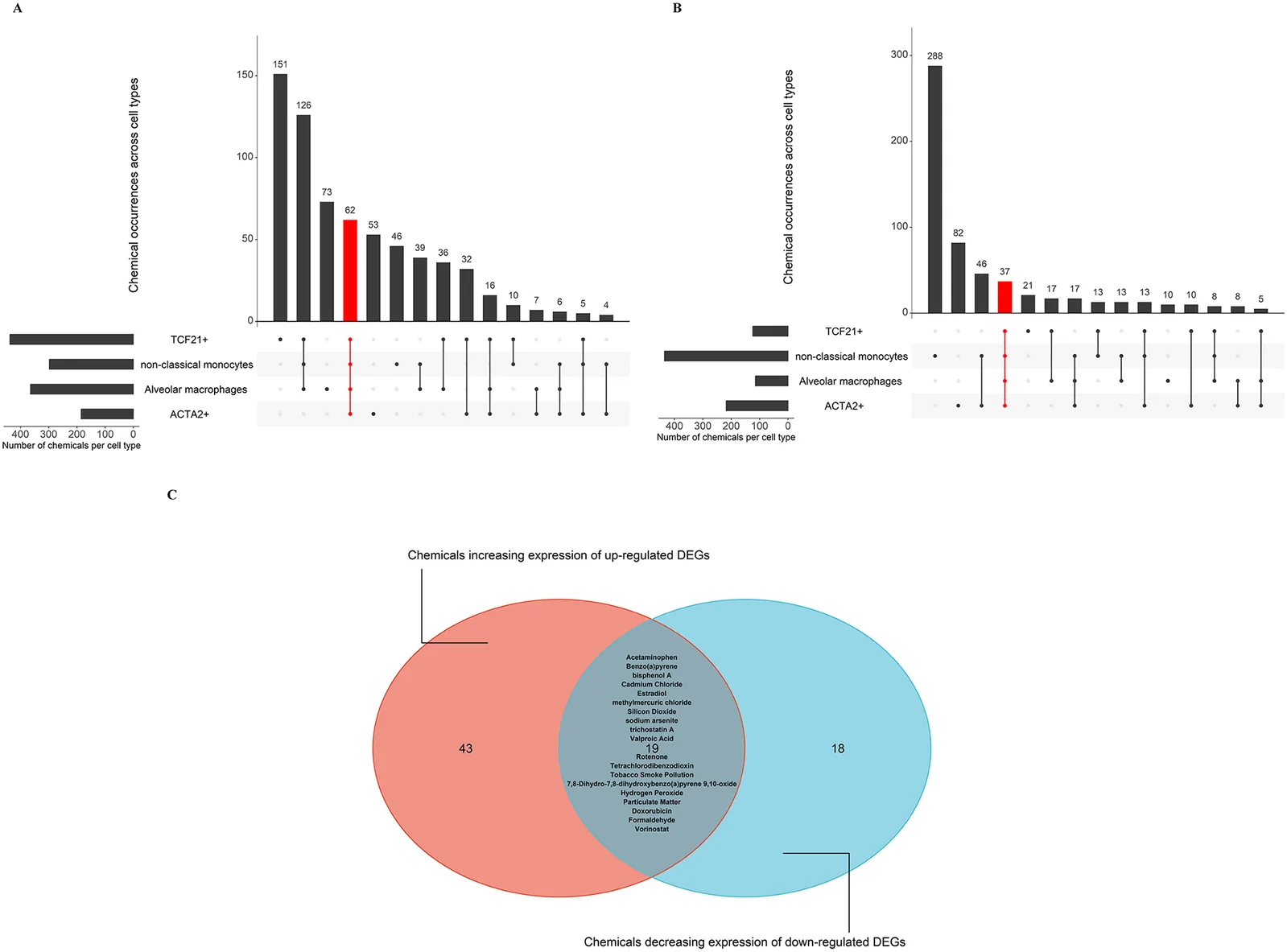
Identification of potential chemical compounds associated with COPD risk. **A**. UpSet plot showing chemical compounds associated with upregulated DEGs in COPD patients compared to healthy controls. **B**. UpSet plot showing chemical compounds associated with downregulated DEGs in COPD patients compared to healthy controls. **C**. Venn diagram illustrating the overlap between chemical compounds associated with upregulated DEGs (left circle) and those associated with downregulated DEGs (right circle).

## 4. Discussion

By integrating multiple publicly available scRNA-seq datasets, this study systematically analyzed the molecular characteristics and intercellular interactions between COPD patients and healthy controls at single-cell resolution. We identified four major cell types and 17 corresponding cell subtypes. Further analyses demonstrated substantial remodeling of cellular communication networks in COPD, particularly affecting signaling functions of certain myeloid cells (mainly macrophages) and fibroblasts. These findings provide new insights into COPD pathogenesis.

Compared with healthy controls, the proportions of immune cell populations differed in COPD patients, with changes observed in macrophage and monocyte subpopulations, suggesting that COPD is associated with remodeling of the inflammatory immune microenvironment. Traditional studies have often analyzed monocytes and macrophages as a relatively homogeneous population. However, through subgroup analysis, our study suggests divergent transcriptional features between alveolar macrophages and non-classical monocytes in COPD. Alveolar macrophages showed enrichment of protein folding, proteostasis-associated processes, and TNF-related inflammatory stress responses, which is consistent with previous evidence linking cellular stress responses to COPD-related inflammation. In contrast, non-classical monocytes showed reduced chemotaxis- and migration-related transcriptional features, including leukocyte migration, neutrophil chemotaxis, and chemokine-response-related processes [45, 46]. These findings suggest that myeloid subpopulations in COPD may undergo heterogeneous immune-state remodeling, in which some subsets display inflammation-associated transcriptional activity whereas others show reduced immune-cell recruitment or migration-related features.

Furthermore, the heterogeneity of fibroblast subpopulations suggests that TCF21 + fibroblasts may be associated with apoptosis-related regulation, complement activation, and immune-response-related transcriptional features. In contrast, ACTA2 + fibroblasts exhibited distinct enrichment of extracellular matrix organization, collagen fibril organization, collagen metabolic process, and cell-substrate adhesion, indicating a matrix-remodeling-associated transcriptional profile. Meanwhile, the downregulated genes in ACTA2 + fibroblasts were enriched in biological processes related to apoptotic signaling regulation, fibroblast growth factor response, mesenchymal cell proliferation, and muscle system regulation, suggesting altered repair- and proliferation-related transcriptional features. Thus, while ACTA2 + fibroblasts may be associated with matrix deposition and remodeling, they also show changes in transcriptional programs related to tissue repair and cellular regulation. These findings provide additional insight into fibroblast heterogeneity in COPD. These cell subpopulations and their specific signaling pathways may serve as candidate cellular and molecular features for future studies of COPD phenotyping and mechanism-oriented validation. For example, proteostasis-associated transcriptional features may help inform future studies of alveolar macrophage-associated inflammatory responses, while ECM remodeling and collagen organization-related pathways may provide clues for investigating fibrosis-associated airway remodeling in COPD.

In addition, chemical–gene interaction analysis identified 19 chemical substances with bidirectional regulatory potential, demonstrating complex and diverse roles in gene expression regulation. Among them, 14 compounds—including tobacco smoke pollution, estradiol, particulate matter, bisphenol A, and sodium arsenite—have already been confirmed to participate in the pathogenesis of COPD [47–58]. For example, a well-recognized environmental risk factor for COPD is exposure to toxic gases and particles from cigarette smoke and biomass burning. Furthermore, estradiol may enhance the susceptibility of lung tissue to oxidative damage caused by smoking by upregulating cytochrome P450 enzyme expression [59]. Long-term exposure to bisphenol A can induce significant inflammatory and oxidative stress responses, characterized by increased malondialdehyde (MDA) levels, reduced superoxide dismutase (SOD) concentration, and upregulated expression of interleukin-18 (IL-18) in lung tissue, thereby influencing COPD progression [60]. Additionally, the roles of five other chemical substances—Trichostatin A, Methylmercuric chloride, Rotenone, Tetrachlorodibenzodioxin, and 7,8-Dihydro-7,8-dihydroxybenzo(a)pyrene 9,10-oxide—in COPD remain unclear, and future research is needed to explore their potential pathophysiological effects. The associations identified in this study should be regarded as broad biological references. Although annotation bias may influence data retrieval, the identification of specific pharmacological agents (e.g., Trichostatin A) alongside common pollutants supports the specificity of our analysis. These findings should be interpreted as exploratory clues regarding potential chemical exposures or regulatory factors, with their biological relevance requiring assessment within the specific context of pulmonary pathology.

This study systematically elucidated the molecular features and intercellular interaction networks in COPD patients at the single-cell level, clarifying the collaborative roles of myeloid and fibroblast subtypes in disease progression. These findings may provide candidate transcriptomic features for future studies of COPD subtype characterization and generate hypotheses for investigating environmental exposure-related mechanisms and chemical-gene associations. Despite the systematic integration of multiple publicly available single-cell sequencing datasets in this study, certain limitations remain, such as the lack of clinical sample information, which prevents adequate consideration of factors such as sex and age on gene expression. It is necessary to combine longitudinal clinical cohort studies with functional experiments to further validate the findings in the future. In addition, further research is needed to translate these molecular mechanisms into clinically applicable intervention strategies and to explore their potential for improving the therapeutic landscape of COPD.

## Supporting information

S1 Data Differentially expressed genes in alveolar macrophages. (XLSX)

S2 Data Differentially expressed genes in non-classical monocytes. (XLSX)

S3 Data GO enrichment of upregulated genes in alveolar macrophages. (XLSX)

S4 Data GO enrichment of downregulated genes in alveolar macrophages. (XLSX)

S5 Data GO enrichment of upregulated genes in non-classical monocytes. (XLSX)

S6 Data GO enrichment of downregulated genes in non-classical monocytes. (XLSX)

S7 Data Differentially expressed genes in TCF21 + cells. (XLSX)

S8 Data Differentially expressed genes in ACTA2 + cells. (XLSX)

S9 Data GO enrichment of upregulated genes in TCF21 + cells. (XLSX)

S10 Data GO enrichment of downregulated genes in TCF21 + cells. (XLSX)

S11 Data GO enrichment of upregulated genes in ACTA2 + cells. (XLSX)

S12 Data GO enrichment of downregulated genes in ACTA2 + cells. (XLSX)

S13 Data Chemical compounds associated with upregulated genes. (XLSX)

S14 Data Chemical compounds associated with downregulated genes. (XLSX)

S15 File Supplementary figures and methods for single-cell analysis of COPD. (DOCX)
