# Supplementary material for: Single-cell analysis of COPD: Microenvironmental interactions driven by immune-fibrotic imbalance
Source: PLoS One. 2026 Oct 26;21(10):e0361580. doi: 10.1371/journal.pone.0361580 (PMC13704830; doi:10.1371/journal.pone.0361580)
Supplement: Supplementary file 1 [file pone.0361580.s015.docx]

**Supplementary Information of**

**Single-Cell Analysis of COPD: Microenvironmental Interactions Driven by Immune-Fibrotic Imbalance**

**Supplementary Figures**

Supplementary Figures 1. Heatmap of ECM-related gene expression in fibroblast subpopulations (healthy control).

Supplementary Figures 2. UMAP visualization of raw data before batch effect correction

Supplementary Figures 3.Raw UMAP projections of three independent datasets prior to batch correction.

Supplementary Figures 4. UMAP projection following Harmony batch effect correction.

Supplementary Figures 5. Harmony-corrected UMAP projections of three independent datasets following batch correction.

Supplementary Figures 6. Dot plot of canonical marker genes across identified cell types.

Supplementary Figures 7. Dot plot of canonical marker genes across identified myeloid cell subtypes.

Supplementary Figures 8. Dot plot of canonical marker genes across identified fibroblast subtypes.

**Supplementary Methods**

Supplementary Methods 1. Parameter settings for dimensionality reduction, clustering, and subpopulation analysis.

Supplementary Methods 2. Intercellular Communication Analysis.

Supplementary Methods 3. Gene Ontology (GO) Enrichment Analysis.

Supplementary Methods 4. Doublet Detection.

Supplementary Methods 5. Chemical-Gene Interaction Analysis.

**Supplementary Figures**

**Supplementary Figure 1: Heatmap of ECM-related gene expression in fibroblast subpopulations (healthy control)**

Supplementary Figure S1 demonstrates a heat map of the expression of genes related to the extracellular matrix (ECM) in a subset of fibroblasts from healthy control samples.Different colors indicate the level of gene expression to reveal the heterogeneity in ECM gene expression in each subpopulation.

**Supplementary Figure 2. UMAP visualization of raw data before batch effect correction.**

UMAP plot displays the distribution of single cells based on raw gene expression profiles prior to batch correction. Cells are colored by their original sample identity (orig.ident), representing 3 distinct samples (Red, Green, Blue).

**Supplementary Figure 3. Raw UMAP projections of three independent datasets prior to batch correction.**

This figure displays the UMAP visualization of single cells pooled from three independent scRNA-seq datasets. Cells are colored by their dataset of origin.

**Supplementary Figure 4. UMAP projection following Harmony batch effect correction.**

This figure presents the UMAP visualization of the dataset after integration using the Harmony algorithm. In contrast to the uncorrected data, cells from the three independent datasets are well-mixed within each cluster, indicating that Harmony has successfully removed technical batch effects while preserving biological structure.

**Supplementary Figure 5. Harmony-corrected UMAP projections of three independent datasets following batch correction.**

Split views of the same UMAP embedding showing the distribution of cells from each independent dataset separately. The consistency of the global structure across all three datasets confirms that the Harmony algorithm has successfully corrected batch differences.

**Supplementary Figure 6. Dot plot of canonical marker genes across identified cell types.**

The dot plot visualizes the expression patterns of selected marker genes (y-axis) used to annotate cell clusters (x-axis). The size of each dot corresponds to the percentage of cells within a cluster expressing the gene, while the color intensity represents the average scaled expression level. The specific enrichment of canonical marker genes in their respective clusters confirms the accuracy of the cell type identification.

**Supplementary Figure 7. Dot plot of canonical marker genes across identified myeloid cell subtypes.**

The specific expression patterns of canonical marker genes validate the accuracy of the annotation for specific myeloid subtypes, such as macrophages.

**Supplementary Figure 8. Dot plot of canonical marker genes across identified fibroblast subtypes.**

The distinct expression patterns of lineage marker genes support the classification of fibroblast subtypes.

**Supplementary Methods**

**Supplementary Methods 1. Parameter settings for dimensionality reduction, clustering, and subpopulation analysis.**

To determine the optimal dimensionality for downstream analyses, we evaluated principal components (PCs) using both the elbow plot and the cumulative variance contribution of each PC. To ensure sufficient biological information and achieve information saturation, we selected the first 40 PCs (PCs 1–40) as input for subsequent neighbor graph construction and clustering.

When constructing the cell-to-cell k-nearest-neighbor (KNN) graph, we set the neighbor parameter k to 20 and then derived the shared-nearest-neighbor (SNN) graph for graph-based clustering.

To identify the best clustering granularity, we performed a multi-resolution scan from 0.1 to 2.0 (step = 0.1) and visualized cluster-tree stability at each resolution with the clustree R package. A hierarchical strategy was adopted with the following parameters:

(a)Global annotation: Taking both cluster stability and the biological characteristics of major lineages into account, we chose resolution 0.6 for primary clustering, which yielded 17 major cell subtypes that were confidently identified and annotated.

(b)Sub-clustering: To dissect intra-lineage heterogeneity further, we re-clustered selected cell populations. Myeloid cells were sub-clustered at a higher resolution of 1.8 to resolve fine-grained subpopulations, whereas fibroblasts were sub-clustered at resolution 0.8. These resolution choices ensured robust cluster structure and clear biological interpretability.

**Supplementary Methods 2. Intercellular Communication Analysis**

Cell-cell communication networks were inferred using the CellChat R package to identify significant ligand-receptor interactions. We utilized the official CellChatDB.human database, which is manually curated from public resources (including FANTOM5, IUPHAR/BPS, and DLRP). This database encompasses three major types of interactions: Secreted Signaling, Cell-Cell Contact, and ECM-Receptor interactions.

To ensure the robustness of communication prediction, strict filtering criteria were applied. Initially, cell groups with fewer than 10 cells were filtered out using the default parameter (min.cells = 10). Significant interactions were identified based on a statistical threshold of p<0.05 using the default permutation test.

**Supplementary Methods 3. Gene Ontology (GO) Enrichment Analysis.**

To functionally characterize specific cell subtypes, Gene Ontology (GO) enrichment analysis was performed using the clusterProfiler R package. Differentially expressed genes (DEGs) were first stratified into up-regulated (avg_log2FC>0) and down-regulated (avg_log2FC<0) groups based on fold change. Gene symbols were converted to Entrez IDs using the org.Hs.eg.db database, with the background universe defined as all genes in the database with valid Entrez ID mappings.

The analysis covered three sub-ontologies: Biological Process (BP), Cellular Component (CC), and Molecular Function (MF) (ont = "all"). To ensure statistical robustness, a minimum gene set size of 10 (minGSSize = 10) was imposed. P-values were adjusted using the Benjamini-Hochberg (BH) method to control the false discovery rate. Significant terms were identified using a dual threshold: nominal p-value<0.05. The top 10 significantly enriched terms per ontology were visualized using dot plots, ranked by GeneRatio, with color indicating statistical significance.

**Supplementary Methods 4. Doublet Detection**

Potential doublets were identified and removed independently for each sample using the DoubletFinder R package (v3). Prior to doublet detection, each dataset underwent standard pre-processing, including normalization, identification of the top 2,000 highly variable features using the vst method, and scaling. Principal Component Analysis (PCA) was performed, and the top 20 PCs were utilized for the subsequent DoubletFinder steps.

To optimize the neighborhood size parameter (*pK*), a parameter sweep was conducted on the top 20 PCs. The optimal pK value was determined for each sample by selecting the value that maximized the Mean-Variance-Normalized Bimodality Coefficient (BCmvn) using the find.*pK* function. The number of expected doublets (*nExp*) was estimated assuming a doublet formation rate of 7% (calculated as 0.07×total cell count). The artificial doublet proportion parameter (*pN*) was set to the default value of 0.25. Cells classified as "Doublets" by DoubletFinder were excluded from downstream analyses.

**Supplementary Methods 5. Chemical-Gene Interaction Analysis**

To identify potential environmental or chemical modulators associated with the observed transcriptomic changes, we performed a chemical-gene interaction analysis using the Comparative Toxicogenomics Database (CTD, accessed February 2025).

(a) Gene Selection and Ranking:

To minimize the risk of false positives driven by noise, a strict set of high-confidence differentially expressed genes (DEGs) was first defined using a significance threshold of Benjamini-Hochberg (BH) adjusted P-value<0.05. Within this significant set, genes were ranked based on the absolute magnitude of their log2 fold change (|log2FC|). To prioritize the most biologically relevant targets, we specifically selected the top 10 up-regulated genes (highest log2FC) and the top 10 down-regulated genes (lowest log2FC) from each cell type as the input signature for chemical interrogation.

(b) Directional Consistency Filtering:

A rigorous "directional consistency" filter was applied as a core rule to screen for key chemicals. We queried the CTD for chemicals known to modulate the expression of the selected DEGs. To reduce false-positive interpretations, we only retained interactions where the direction of chemical modulation matched the observed gene expression change:

- For up-regulated genes, only chemicals with curated evidence of promoting/increasing expression were retained.
- For down-regulated genes, only chemicals with evidence of inhibiting/decreasing expression were kept.

(c) Consensus Analysis:

Finally, to ensure the robustness of the identified candidates, we calculated the intersection of chemical lists derived from different cell types (or analysis groups). Only chemicals consistently present across these intersections were defined as the final key chemical candidates.
